# Long noncoding RNA LINP1: scaffolding non-homologous end joining

**DOI:** 10.1038/cddiscovery.2016.59

**Published:** 2016-07-18

**Authors:** A Sakthianandeswaren, S Liu, OM Sieber

**Affiliations:** 1 Systems Biology and Personalised Medicine Division, The Walter and Eliza Hall Institute of Medial Research, Parkville, VIC, Australia; 2 Department of Medical Biology, The University of Melbourne, Parkville, VIC, Australia; 3 Department of Surgery, The University of Melbourne, Parkville, VIC, Australia; 4 School of Biomedical Sciences, Monash University, Melbourne, VIC, Australia

Non-homologous end joining (NHEJ) is a principal pathway of DNA double-strand break (DSB) repair in mammalian cells,^1^ but is also involved in assembly of antigen receptor genes and telomere maintenance.^2,3^ Loss of NHEJ function can result in chromosome instability promoting malignant transformation, in particular when other cellular safeguards are compromised. Inherited NHEJ defects underlie radiosensitive severe combined immunodeficiency. NHEJ is initiated by binding of Ku70–Ku80 heterodimers to both ends of a DNA break, followed by recruitment of catalytic subunits of DNA-dependent protein kinase (DNA-PKcs) to form juxtaposed DNA-PK holoenzymes which join the ends in a synaptic complex. Cohesive blunt DNA ends can be joined directly by the XFL-XRCC4-DNA ligase IV complex, but in many cases DNA ends contain damaged bases or DNA backbone sugars that require processing before ligation. End processing may involve nucleases such as Artemis, DNA polymerases and polynucleotide kinase among other enzymes.^4,5^

In Nature Structural & Molecular Biology, Zhang *et al.*^6^ recently reported the discovery of a long noncoding RNA (lncRNA) termed lncRNA in non-homologous end joining (NHEJ) pathway 1 (LINP1), that serves as an RNA scaffold interacting with Ku70–Ku80 and DNA-PKcs, promoting NHEJ repair (Figure 1). LINP1 was identified as overexpressed in triple-negative breast cancers (TNBCs) when compared with other breast cancer subtypes using RNA-seq data from The Cancer Genome Atlas and the Cancer Cell Line Encyclopedia (CCLE). LINP1 knockdown enhanced apoptosis in TNBC cell lines following doxorubicin treatment, a chemotherapy drug for TNBC, while overexpression of LINP1 in an estrogen-receptor positive breast cancer (ER+ BC) cell line with undetectable LINP1 protected these cells from doxorubicin-induced apoptosis.

Using RNA pull-down, CHART and RNA-IP assays, Zhang *et al.*^6^ demonstrated that LINP1 interacted with Ku70–Ku80 and DNA-PKcs. Specifically, LINP1 transcript bound to Ku80 via its 5′ region, and to DNA-PKcs via its 3′ region. LINP1 knockdown in TNBC cells resulted in reduced DNA break repair in comet, γ-H2AX foci and NHEJ reporter assays. Conversely, overexpression of LINP1 in ER+ BC cells increased NHEJ activity. Analyses of chromatin-associated NHEJ complexes post-irradiation with LINP1, Ku80 or DNA-PKcs knockdown indicated that recognition of DSBs by Ku70–Ku80 resulted in recruitment of LINP1 and DNA-PKcs, with LINP1 enhancing the Ku80–DNA-PKcs interaction. It will be interesting to determine the protein domains in Ku80 and DNA-PKcs mediating LINP1 interaction, whether LINP1 binding is regulated by or affects post-translational modifications and conformation states of Ku70–Ku80 or DNA-PKcs, and to what extent this impacts on other NHEJ components.

EGF signaling was identified as a positive regulator of LINP1 expression. LINP1 levels were correlated with EGFR expression in primary breast cancers and CCLE cell lines. In CCLE cell lines, LINP1 expression was associated with the EGF downstream transcription factors c-Jun and c-Fox (both AP1 transcription factors), suggesting regulation via the RAS–MEK–JNK pathway. Accordingly, EGF treatment induced LINP1 in TNBC cells, but not in ER+ BC cells and LINP1 expression in TNBC cell lines could be repressed with EGFR, MEK and JNK inhibitors. ENCODE ChIP-seq data indicated enrichment of c-Jun or c-Fos at the LINP1 promoter in TNBC cells, validated by ChIP-qPCR with increased binding following EGF stimulation. An AP1-binding site in the LINP1 promoter was required for EGF activation, and this could be blocked with EGFR, MEK and JNK inhibitors. It is intriguing to speculate that EGFR amplification or oncogenic mutations in RAS or RAF may enhance NHEJ activity by upregulating LINP1, perhaps contributing to their pro-tumorigenic roles by increasing resistance to genotoxic insults. Notably, EGF signaling has other established links to NHEJ, with binding of EGFR to DNA-PK enhancing NHEJ activity.^7^

LINP1 was shown to be repressed by p53 signaling. In primary breast cancers and cell lines, LINP1 was elevated in *TP53*-mutated cases. The MDM2 inhibitor nutlin-3a repressed LINP1 in *TP53* wild-type but not mutated TNBC cells, a finding replicated in *TP53* isogenic colon cancer cells. The LINP1 promoter was non-responsive to p53 expression. However, two regions in LINP1 exon 2 were complementary to the seed sequence of miR-29, a downstream target of p53. miR-29 expression increased on nutlin-3a treatment in *TP53* wild-type but not mutant or null cells. Transduction of miR-29 mimic into TBNC and isogenic colon cancer cells resulted in LINP1 downregulation irrespective of genotype, and repressed luciferase reporter expression for wild-type LINP1, but not a LINP1 with a miR-29 seed-sequence mutation. Based on the observation that LINP1 enhancement of NHEJ activity occurred immediately after DNA damage, while the miR-29-mediated LINP1 downregulation occurred at a later time point, the authors proposed that p53–miR-29 may serve as a negative feedback mechanism to restrict NHEJ activity. While the current model suggests reduced miR-29 expression in *TP53*-mutated TBNC, this remains to be validated in primary cancers.

Besides p53 signaling identified by Zhang *et al.*,^6^ multiple other pathways have previously been implicated in miR-29 regulation including suppression by MYC, PU.1, NF-κB and TGF-β, and induction by GATA3, MBP-1 and Sox2. miR-29 is ectopically expressed in various cancers.^8^ Perhaps under these conditions, miR-29 could play a role in compromising NHEJ via repressing LINP1, potentially promoting chromosome instability.

NHEJ pathway activity is an established determinant of radiation (IR) and chemotherapy resistance in cancer,^9^ with multiple trials targeting the pathway presently ongoing. Accordingly, LINP1 modulated IR sensitivity in breast cancer cells. LINP1 knockdown decreased survival after IR treatment in TNBC cell lines *in vitro*, while overexpression of LINP1 in ER+ BC cells rendered these more resistant. Similarly, LINP1 knockdown in a xenograft model of TNBC cells attenuated tumor growth and was associated with increased γ-H2AX foci after DNA damage. Negative regulation of LINP1 levels by miR-29 may highlight an alternative way of therapeutic targeting of the NHEJ pathway through the use of miR-29 mimetics.

The study clearly identified TNBCs as exhibiting elevated levels of LINP1 expression when compared with other breast cancer subtypes. LINP1 gene copy number gain, *TP53* mutation and EGFR expression were all associated with LINP1 levels. However, it cannot be excluded that other mechanisms linked to the etiology of TNBCs are responsible for high-LINP1 levels. The doxorubicin and IR sensitivity studies of Zhang *et al.*^6^ could be interpreted as implying non-oncogene addiction to the NHEJ pathway in TNBCs, indicating this tumor type as a prime candidate for NHEJ pathway targeting. However, conclusive demonstration that elevated LINP1 levels in TNBCs are a robust indicator of activity and dependence on NHEJ, as compared with tumor types with low-LINP1 expression, will require expanded studies. Relative dependence on NHEJ may also depend on the status of other DNA DSB repair pathways such as *BRCA1* or *BRCA2* mutations which occur in ~10–20% of TNBCs.^10^

Zhang *et al.*^6^ have identified LINP1 as an integral component of the synaptic complex of NHEJ, regulated via EGF and p53 signaling. While their study focused on TNBC, these findings have profound implications for our general understanding of the mechanism of DNA DSB repair by NHEJ. To what extent cellular LINP1 expression levels indicate NHEJ functional status remains to be elucidated. If confirmed, LINP1 could plausibly play a dual role in cancer, with overexpression increasing resistance to genotoxic insults and loss of expression promoting chromosome instability. Direct targeting of LINP1 by miR-29 mimetics may prove a fruitful avenue for therapeutic inhibition of NHEJ in certain clinical contexts.

## Figures and Tables

**Figure 1:**
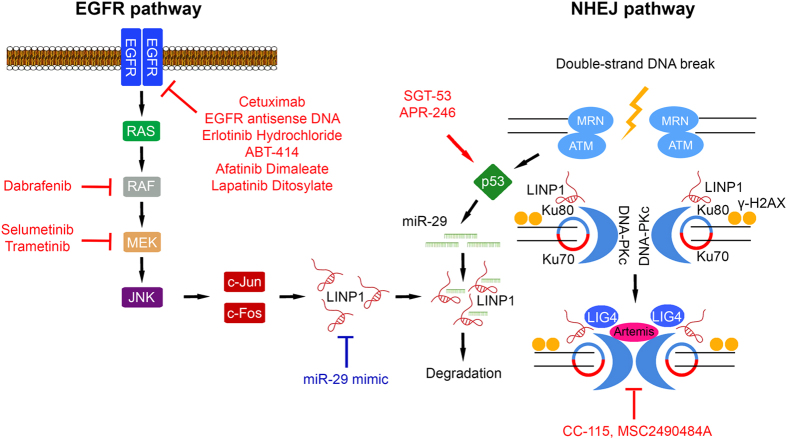
LINP1 regulation and role in the NHEJ DNA-repair pathway. LINP1 expression levels are positively and negatively regulated by the EGF and p53 signaling pathways, respectively. On recognition of DNA DSBs by the Ku70–Ku80 complex, LINP1 and DNA-PKcs are recruited to form a synaptic complex joining the broken DNA ends and initiating DNA repair. NHEJ inhibitors currently evaluated in anti-cancer clinical trials in combination with radiotherapy are indicated, with miR-29 mimetics potentially offering a novel therapeutic avenue.
